# Conditioned Serum Enhances the Chondrogenic and Immunomodulatory Behavior of Mesenchymal Stem Cells

**DOI:** 10.3389/fphar.2019.00699

**Published:** 2019-06-28

**Authors:** Rebeca Blázquez, Francisco Miguel Sánchez-Margallo, Julio Reinecke, Verónica Álvarez, Esther López, Federica Marinaro, Javier G. Casado

**Affiliations:** ^1^Stem Cell Therapy Unit, “Jesús Usón” Minimally Invasive Surgery Centre, Cáceres, Spain; ^2^CIBER de Enfermedades Cardiovasculares (CIBER-CV), Madrid, Spain; ^3^Research and Development Department, ORTHOGEN AG, Düsseldorf, Germany

**Keywords:** autologous conditioned serum, mesenchymal stem cells, chondrogenic, immunomodulation, osteoarthritis, Orthokine

## Abstract

Osteoarthritis is one of the most common chronic health conditions associated with pain and disability. Advanced therapies based on mesenchymal stem cells have become valuable options for the treatment of these pathologies. Conditioned serum (CS, “Orthokine”) has been used intra-articularly for osteoarthritic patients. In this work, we hypothesized that the rich content on anti-inflammatory proteins and growth factors of CS may exert a beneficial effect on the biological activity of human adipose-derived mesenchymal stem cells (hAdMSCs). *In vitro* studies were designed using hAdMSCs cocultured with CS at different concentrations (2.5, 5, and 10%). Chondrogenic differentiation assays and immunomodulatory experiments using *in vitro*-stimulated lymphocytes were performed. Our results demonstrated that CS significantly enhanced the differentiation of hAdMSCs toward chondrocytes. Moreover, hAdMSCs pre-sensitized with CS reduced the lymphocyte proliferation as well as their differentiation toward activated lymphocytes. These results suggest that *in vivo* coadministration of CS and hAdMSCs may have a beneficial effect on the therapeutic potential of hAdMSCs. Moreover, these results indicate that intra-articular administration of CS might influence the biological behavior of resident stem cells increasing their chondrogenic differentiation and inherent immunomodulatory activity. To our knowledge, this is the first *in vitro* study reporting this combination.

## Introduction

Osteoarthritis is one of the most common chronic health conditions associated with pain and disability (Allen and Golightly, 2015), being the most common form of arthritis followed by rheumatoid arthritis, gout, lupus, fibromyalgia, and others (Barbour et al., 2017). Osteoarthritis is a highly prevalent joint disorder worldwide affecting approximately 15% of the population (Johnson and Hunter, 2014), which is mainly characterized by cartilage destruction (Manek and Lane, 2000), affecting to the entire joint structure (Pelletier et al., 2001).

The traditional treatments of osteoarthritis are mainly based on a combination of pharmacological and non-pharmacological options aiming to reduce pain and gain quality of life in the patient (Hunter, 2011). These treatments do not address the cause of the disease being frequently associated to renal, cardiovascular, and gastrointestinal secondary effects mainly due to the use of nonsteroidal anti-inflammatory drugs. In the most severe cases, surgical joint prosthesis implantation is performed, also with serious side effects (Tonge et al., 2014).

At the present, great efforts are being made to develop new therapeutic tools for arthritic diseases. In this sense, platelet-rich plasma (PRP) has emerged in the last years as a promising therapeutic option for cartilage degeneration, demonstrating its chondroprotective effect (Andia and Maffulli, 2013; Moussa et al., 2017) and a temporal pain relief together with a functional improvement of the involved joint (Laver et al., 2017). However, the variation between different preparations makes difficult the establishment of its therapeutic potential (Richards et al., 2016), and postinjection pain seems to be more frequent after PRP intra-articular administration than with other treatments using the same route (Ornetti et al., 2016).

At the end of the 1990s, a method based on the exposure of blood leukocyte to pyrogen-free surfaces (medical-grade glass beads) has become a valuable option for the treatment of these pathologies. This autologous conditioned serum (ACS), firstly described by Meijer et al. in 2003, has shown a high concentration on interleukin 1 receptor antagonist (IL-1Ra) and other anti-inflammatory cytokines and growth factors such as interleukins 4 (IL-4), 6 (IL-6), and 10 (IL-10) epidermal growth factor (EGF), vascular endothelial growth factor (VEGF), hepatocyte growth factor (HGF), insulin-like growth factor 1 (IGF1), platelet-derived growth factor (PDGF), and transforming growth factor beta 1 (TGFβ1) (Meijer et al., 2003; Wehling et al., 2007). As IL-1 is a major contributor to osteoarthritis pathogenesis, stimulating chondrocytes and synovial fibroblasts to upregulate matrix metalloproteases that damage the cartilage, the blockade of this cytokine offers important benefits (Richards et al., 2016). This biological treatment has been assayed in veterinary medicine for the treatment of osteoarthritis in horses (Frisbie et al., 2007). Clinical trials have demonstrated its beneficial effect in knee osteoarthritis (Baltzer et al., 2009; Baselga García-Escudero and Miguel Hernández Trillos, 2015), lumbar radicular compression (Becker et al., 2007; Godek, 2016), supraspinatus pathology (von Wehren et al., 2016), and after anterior cruciate ligament surgery (Darabos et al., 2011) being superior to traditional treatments such as hyaluronan (for osteoarthritis) and triamcinolone (for lumbar radicular compression).

On the other hand, the administration of mesenchymal stem cell (MSC)-based therapies, either locally or systemically, has emerged in the last years as an encouraging therapeutic tool for the treatment of inflammatory-based diseases (de Witte et al., 2015). It has been demonstrated that they can also decrease the adverse effect of IL-1β in osteoarthritis (Jin et al., 2017). In veterinary medicine, intra-articular injections of MSCs have been administered in horses using xenogeneic, allogeneic, and autologous approaches (Pigott et al., 2013). These cells have demonstrated to exert an immunomodulatory effect on cells from the innate and adaptive immune system. More recently, they have shown very promising results in clinical trials addressing the treatment of osteoarticular diseases (Jorgensen and Noël, 2011; Pers and Jorgensen, 2016).

Putting together these new therapies, a review comparing bone marrow aspirates, PRP, and ACS as alternative treatment options for musculoskeletal diseases concluded that while bone marrow aspirates and PRP are successful in treating early arthritis, ACS is a better option for chronic joint arthritis (Patel and Strauss, 2015). In a recent review from Fotouhi et al., different treatments for knee osteoarthritis were discussed from a historical perspective. The authors suggest that PRP, stromal vascular fraction, and ACS seem to have favorable and promising results (Fotouhi et al., 2018).

In this work, we hypothesized that the rich content on anti-inflammatory proteins and growth factors of pooled ACS (CS) may exert a beneficial effect on the biological activity of human adipose-derived MSCs (hAdMSCs). Our results indicate that CS is an appropriate vehicle for MSCs administration, as their viability and proliferative behavior are comparable with standard culture conditions. Moreover, the *in vitro* experiments showed that CS induces the chondrogenic differentiation of hAdMSCs and enhances their immunomodulatory potential. This study suggests that CS sensitization may exert a beneficial effect on the therapeutic potential of hAdMSCs that needs to be further addressed in an appropriate *in vivo* model to confirm these results. To our knowledge, this is the first *in vitro* study reporting this combination.

Finally, it is important to note that our *in vitro* observations could be translated to the clinical situation when ACS is intra-articularly administered. In this sense, the induction of chondrogenic differentiation and the enhancement of the immunomodulatory potential of articular resident stem cells could be triggered by the administration of ACS.

## Material and Methods

### Collection of Human Conditioned Serum

Human CS was obtained from three volunteers as previously described (Wehling et al., 2007) by incubating 10 ml of venous blood in the presence of medical-grade glass beads (Orthogen, Düsseldorf, Germany) for 6 h at 37°C to ensure physiological conditions. These glass beads induce the production of anti-inflammatory cytokines (IL-1Ra, IL-10, and IL-6) and growth factors (EGF, PDGF, TGF-β1, VEGF, HGF, and IGF-1) by white blood cells. After incubation, the blood-filled syringes were centrifuged at room temperature in a universal compact centrifuge (Hermle Z 200 A) in a fixed angle rotor at 2,100 g for 10 min. The serum supernatant was filtered by Millex GP 0.22 µm syringe tip filter, pooled, filtered again, aliquoted, and stored at −20°C until use. The biological samples were obtained after written informed consent under the auspices of the appropriate research and ethics committees and in accordance with the Declaration of Helsinki. This study was approved by Minimally Invasive Surgery Centre Research Ethics Committee (approval number: SITC215).

### Isolation and Expansion of Human Adipose Mesenchymal Stem Cells

The human adipose-derived mesenchymal stem cells (hAdMSCs) were obtained from lipoaspirated human adipose tissue from healthy adult donors as previously described (DelaRosa et al., 2012). Briefly, tissue was washed with phosphate-buffered saline (PBS) solution and digested at 37°C for 30 min with 0.075% collagenase type I (Invitrogen, Carlsbad, CA, USA) in PBS. Samples were then washed with 10% fetal bovine serum (FBS), incubated with ammonium chloride 160 mM at room temperature for 10 min to lyse red blood cells, suspended in Dulbecco’s modified Eagle’s medium (DMEM) containing 10% FBS, and filtered by a 40 μm nylon mesh. After cell seeding, hAdMSCs were expanded at 37°C and 5% CO_2_, with medium replacement every 7 days. When 90% of confluence was reached, cells were detached with a 0.25% trypsin solution and seeded again at a density of 5,000 cells/cm^2^ into new culture flasks to continue cell expansion. Three cell lines from different healthy donors were used for subsequent experiments. The biological samples were obtained after written informed consent under the auspices of the appropriate Research and Ethics Committees and in accordance with the Declaration of Helsinki. This study was approved by Minimally Invasive Surgery Centre Research Ethics Committee (approval number: SITC215).

### Phenotypic Analysis

For flow cytometric analysis by fluorescence-activated cell sorting (FACS), the hAdMSCs were cultured in the presence of CS or FBS (GE Healthcare Hyclone, GE Healthcare, Chicago, IL, USA) at different concentrations (2.5, 5, and 10%) in the culture medium (DMEM supplemented with 1% penicillin/streptomycin and 1% glutamine). After 6 days, cells were trypsinized and labeled with fluorescein isothiocyanate (FITC)-conjugated human monoclonal antibodies (mAbs) against CD44, CD45, CD90, and human leucocyte antigen DR (HLA-DR), and phycoerythrin (PE)-conjugated human mAb against CD73 (BD Biosciences, San Jose, CA, USA). After incubating 200,000 cells with manufacturer’s suggested concentration of mAbs in PBS with 2% FBS at 4°C for 30 min, cells were washed with PBS and resuspended again. A FACScalibur cytometer (BD Biosciences, San Jose, CA, USA) was used for the cytometric analysis. A total of 100,000 events were acquired, and a selection based on forward and side scatter properties was performed before fluorescence analysis with CellQuest software (BD Biosciences, San Jose, CA, USA). Mean relative fluorescence intensity was obtained by dividing the mean fluorescent intensity by the mean fluorescent intensity of its negative control (isotype-matched antibodies).

### Cell Proliferation Assays

Cell proliferation was determined using Cell Counting Kit 8 (CCK-8), which measures the activity of living cells by assessing their mitochondrial activity. The reaction product is directly proportional to the number of living cells and can be spectrophotometrically quantified. To perform this determination, cells were seeded at a density of 5,000 cells/well in 96-well plates in DMEM medium with different concentrations (2.5, 5, and 10%) of CS or FBS. The proliferative activity was measured at 0 and 6 days. For that, the culture medium was replaced by 200 μl of phenol-red free DMEM, and 20 μl of CCK-8 (WVR, Radnor, PA, USA) were added. The absorbance of the supernatants was read 2 h later at 450 nm.

### Viability Determination

The viability of the hAdMSCs cultured for 6 days in standard culture conditions (10% FBS) and with different concentrations (2.5, 5, and 10%) of CS was calculated by a trypan blue dye-exclusion assay using the Countess^®^ Automated Cell Counter (Thermo Fisher Scientific Inc., Waltham, MA, USA).

### Chondrogenic Differentiation Assay

For the chondrogenic differentiation assay, hAdMSCs at 80% of confluence with CS or FBS at different concentrations (2.5, 5, and 10%) were cultured for 15 days in standard culture medium (DMEM supplemented with 1% penicillin/streptomycin and 1% glutamine), with or without StemPro Chondrogenesis Supplement (Thermo Fisher Scientific, Gibco™) and replacing the medium every 3 days. To evidence the chondrogenic differentiation, cultured cells were fixed, and an Alcian Blue 8GX staining was performed, as recommended by the International Society of Cellular Therapy (Dominici et al., 2006). To quantify the differentiation degree, cells were lysed with 6 M guanidine–HCl to extract the colorant, and the absorbance of extracts was read at 600 nm.

### Active and Latent TGF-β Determination on Conditioned Serum

The quantification of active and latent TGF-β1 in CS was performed by emzyme-linked immunosorbent assay (ELISA) using the LEGEND MAX™ Free active TGF-β1 and LEGEND MAX™ Latent TGF-β1 ELISA kits (Biologend, San Diego, CA, USA) according to manufacturer’s instructions. Active and latent TGF-β1 were directly measured in CS and in a 1:10 dilution (CS in DMEM).

### Lymphocytes Isolation and Preservation

Peripheral blood lymphocytes (PBLs) were isolated from peripheral blood samples of three healthy donors obtained after written informed consent under the auspices of the appropriate research and ethics committees and in accordance with the Declaration of Helsinki. Peripheral blood was diluted in PBS and centrifuged over Histopaque-1077 (Sigma, St. Louis, MO, USA) at 400 g for 20 min with brakes off. PBL-containing fraction was recovered, washed twice with PBS, and resuspended in FBS with 10% dimethyl sulfoxide (DMSO) for liquid nitrogen preservation. For thawing, aliquots were submerged in 37°C water and diluted in 10 ml Roswell Park Memorial Institute (RPMI) medium 1640. Subsequent centrifugation at 1,500 rpm for 5 min was performed to eliminate DMSO remnants. Cell pellet was resuspended in DMEM and used for experiments.

### *In Vitro* Stimulation of T Cells and Coculture With Conditioned Serum-Sensitized Human Adipose-Derived Mesenchymal Stem Cells

To determine the immunomodulatory effect of CS-sensitized hAdMSCs on *in vitro*-stimulated PBLs, a previously described protocol was used (Blazquez et al., 2014). Briefly, 200 μl of a 1 × 10^6^ PBLs/ml suspension was seeded per well in 96-well plates. A T cell activation/expansion kit (Miltenyi Biotec Inc, San Diego, CA, USA) was used to stimulate PBLs, following the manufacturer’s recommendations. Stimulated PBLs were cocultured for 6 days in contact with previously sensitized hAdMSCs. This sensitization was performed after seeding 5,000 hAdMSCs/well in a 96-well plate, by culturing them in the presence of different concentration (2.5, 5, and 10%) of CS or FBS in the culture medium (DMEM supplemented with 1% penicillin/streptomycin and 1% glutamine) for 6 days before the coculture with PBLs in the same culture medium. Negative controls (non-stimulated PBLs) and positive controls (stimulated PBLs without hAdMSCs) were used in all the experiments.

### Carboxyfluorescein Succinimidyl Ester Proliferation Assay

To determine the proliferative behavior of T lymphocytes, these cells were stained with carboxyfluorescein succinimidyl ester (CFSE) using the CellTrace™ CFSE Cell Proliferation Kit (Invitrogen, Eugene, OR, USA) according to manufacturer’s instructions. Briefly, PBLs were incubated for 10 min at 37°C with the reagent at a final concentration of 10 μM. Culture medium (DMEM supplemented with 1% penicillin/streptomycin and 1% glutamine) was then added in order to remove dye remnants. Stained PBLs were cultured in the presence of CS-sensitized hAdMSCs for 6 days as described in “*In Vitro* Stimulation of T Cells and Co-culture With CS-Sensitized hAdMSCs”. These PBLs in suspension were collected from wells and stained with fluorescence-labeled human mAbs against CD4 and CD8 (BD Biosciences, San Jose, CA, USA).

For the fluorescence analysis, 200,000 cells were incubated with manufacturer’s suggested concentration of mAbs in PBS with 2% FBS at 4°C for 30 min, washed with PBS, and resuspended again. The FACScalibur cytometer (BD Biosciences, San Jose, CA, USA) was used for the cytometric analysis. A total of 100,000 events were acquired, and a selection based on forward and side scatter properties was performed before fluorescence analysis with CellQuest software (BD Biosciences, San Jose, CA, USA). Isotype-matched negative control antibodies were used in all the experiments. The percentage of proliferative cells was calculated as the percentage of CFSE-low fluorescence intensity cells on gated CD4+ and CD8+ T cells.

### Differentiation/Activation Markers Expression Analysis on *In Vitro*-Stimulated PBLs

For flow cytometric analysis of *in vitro*-stimulated PBLs, these cells were collected from wells by recovering the supernatant after 6 days of culture in the presence of CS-sensitized hAdMSCs. The cells were stained with fluorescence-labeled human mAbs against CD62L and CD45RA (BD Biosciences, San Jose, CA, USA).

For the fluorescence analysis, 200,000 cells were incubated with manufacturer’s suggested concentration of mAbs in PBS with 2% FBS at 4°C for 30 min, washed with PBS, and resuspended again. The FACScalibur cytometer (BD Biosciences, San Jose, CA, USA) was used for the cytometric analysis. A total of 100,000 events were acquired, and a selection based on forward and side scatter properties was performed before fluorescence analysis with CellQuest software (BD Biosciences, San Jose, CA, USA). Isotype-matched negative control antibodies were used in all the experiments. The percentage of naïve cells was determined as the percentage of CD45RA+/CD62L+ cells on forward scatter (FSC)/side scatter (SSC)-gated cells.

### Statistical Analysis

Data were statistically analyzed using a one-way ANOVA test. When statistically significant differences were found, Fisher’s least significant difference test was performed comparing equivalent concentrations of CS and FBS, C− and C+, and C+ with each treatment group. The *p*-values ≤0.05 were considered statistically significant. All the statistical determinations were made using SPSS-21 software (SPSS Chicago, IL, USA).

## Results

### Phenotypic Profile of Surface Markers on Human Adipose-Derived Mesenchymal Stem Cells Cultured in the Presence of Conditioned Serum

**Figure 1:**
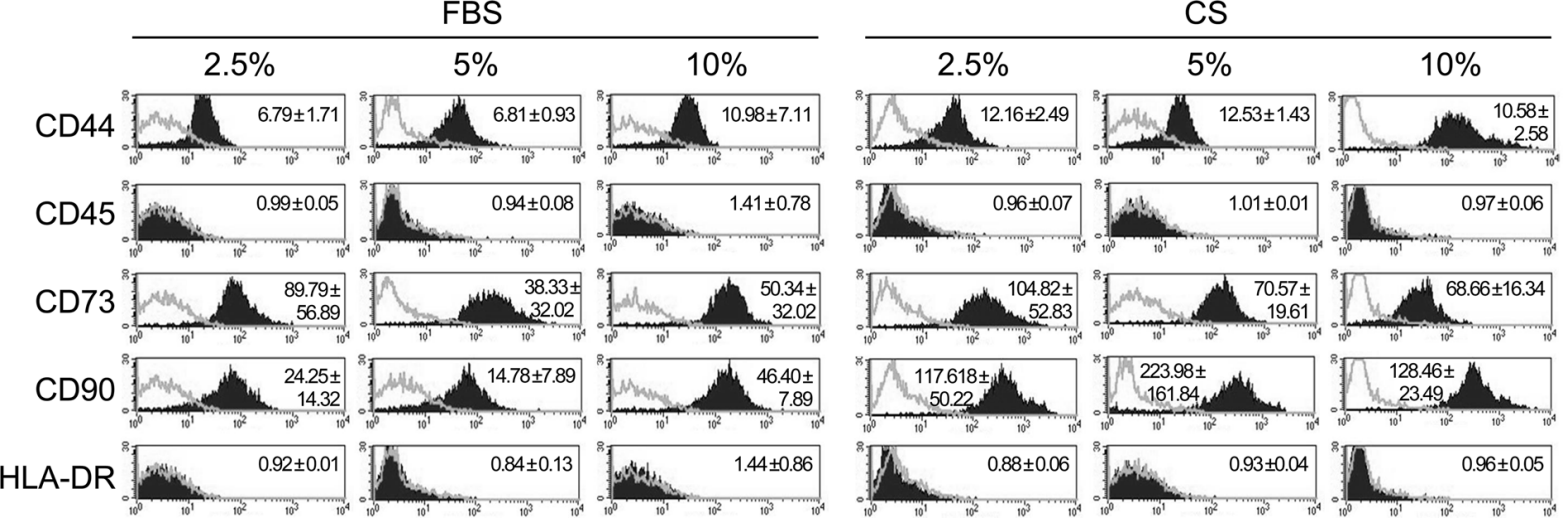
Phenotypic analysis of human adipose-derived mesenchymal stem cells (hAdMSCs) cultured in the presence of fetal bovine serum (FBS) or conditioned serum (CS). Different hAdMSCs cell lines (*n* = 3) were cultured in Dulbecco’s modified Eagle’s medium (DMEM) supplemented with 1% penicillin/streptomycin and 1% glutamine and in the presence of FBS or CS at different concentrations (2.5, 5, and 10%). After 6 days, the presence/absence of different surface markers was analyzed by flow cytometry. Representative histograms together with the relative expression of cell surface markers (mean ± SD) are shown. The relative expression is represented as mean relative fluorescence intensity, which is calculated by dividing the mean fluorescent intensity (filled histogram) by its negative control (gray-lined histogram).

In order to compare the phenotype of hAdMSCs cultured in the presence of FBS with those cultured in the presence of CS, the expression of stem cell markers was analyzed by flow cytometry. The stem cell phenotype of hAdMSCs cultured with 2.5, 5, and 10% of FBS or CS was determined after 6 days by flow cytometry using commercially available mAbs against CD44, CD45, CD73, CD90, and HLA-DR. Our results showed that the phenotype profiles of hAdMSCs under different conditions were similar. As shown in **Figure 1**, the surface marker expression was CD44+/CD45-/CD73+/CD90+/HLA-DR- for both culture conditions. However, CD90 expression was more pronounced on hAdMSCs cultured with CS.

### Proliferative Ability of Human Adipose-Derived Mesenchymal Stem Cells Cultured in the Presence of Conditioned Serum

**Figure 2:**
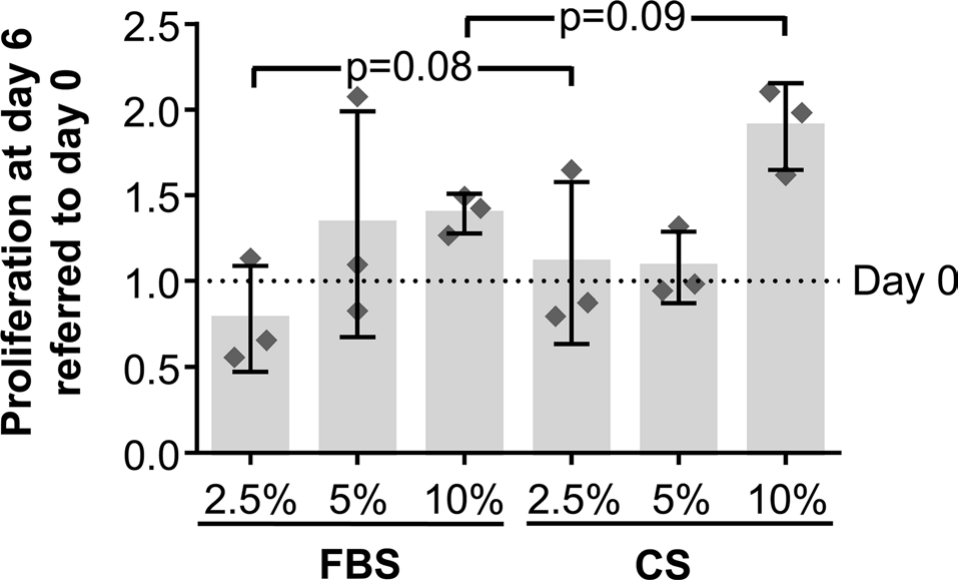
*In vitro* proliferation of hAdMSCs cultured in the presence of FBS or CS. The cells were cultured in DMEM supplemented with 1% penicillin/streptomycin and 1% glutamine and in the presence of FBS or CS at different concentrations (2.5, 5, and 10%). The proliferative behavior of cells was determined through a Cell Counting Kit 8 (CCK-8) assay, where the mitochondrial activity of living cells was spectrophotometrically quantified at 450 nm at days 0 and 6. The graph shows the increase or decrease of absorbance at day 6 normalized and expressed according to absorbance at day 0. Individual values (*n* = 3) as well as mean ± SD are shown. Data were statistically analyzed using a one-way ANOVA test followed by a Fisher’s least significant difference test to compare equivalent concentrations of CS and FBS. *p*-values <0.1 are shown.

To determine whether CS has an effect on hAdMSCs’ proliferative behavior, the proliferation rate of hAdMSCs cultured in the presence of FBS or CS (2.5, 5, and 10%) was compared by a CCK-8 assay. The proliferation rate was quantified at day 0 (negative control) and at day 6. Our results showed no significant differences when comparing the proliferative behavior of cells cultured in the presence of different concentrations of FBS or CS. These results demonstrate that the proliferation rate of hAdMSCs was unaffected by the presence of CS. Interestingly, as shown in **Figure 2**, there was an increase, although it does not reach statistical significance (0.05 ≤ *p* ≤ 0.1), in the proliferative rate of hAdMSCs cultured in the presence of 2.5 and 10% CS (when compared with the same concentrations of FBS). Additionally, viability of hAdMSCs cultured in the different conditions was determined by a trypan blue dye-exclusion assay, without any significant difference (**Supplementary Figure 1**).

### Chondrogenic Differentiation of Human Adipose-Derived Mesenchymal Stem Cells Cultured in the Presence of Conditioned Serum

**Figure 3:**
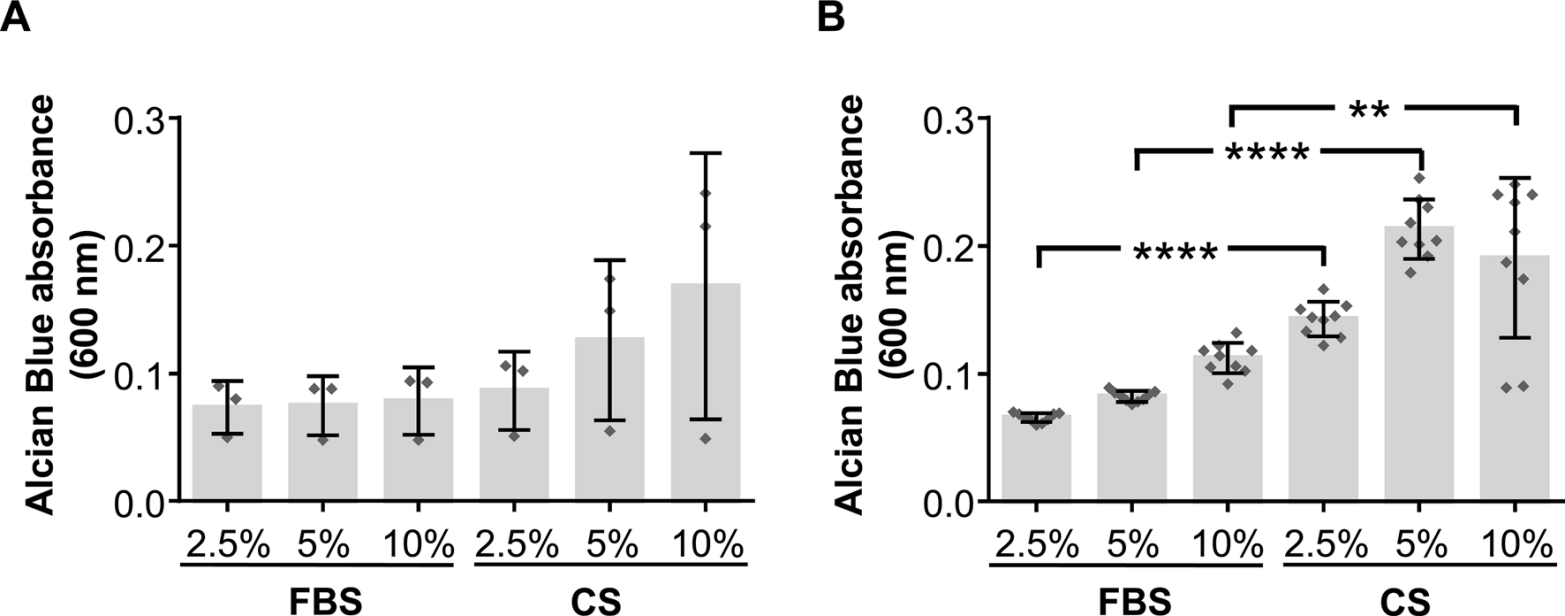
Chondrogenic differentiation potential of hAdMSCs cultured in the presence of FBS or CS. Cells were cultured in the presence of FBS or CS at different concentrations (2.5, 5, and 10%) for 15 days in standard medium (DMEM supplemented with 1% penicillin/streptomycin and 1% glutamine) as negative control **(A)** or in chondrogenic differentiation specific medium **(B)**. Chondrogenic differentiation was confirmed by Alcian Blue 8GX staining that evidences the presence of accumulated glycosaminoglycans. For the quantification of the differentiation degree, cells were lysed to release the colorant, which was spectrophotometrically measured at 600 nm. Graphs represent individual values (**A**, *n* = 3; **B**, *n* = 9) as well as mean ± SD of three independently performed experiments. Data were statistically analyzed using a one-way ANOVA test followed by a Fisher’s least significant difference test to compare equivalent concentrations of CS and FBS. **p ≤ 0.01; ****p ≤ 0.0001.

In order to assess the chondrogenic differentiation potential of hAdMSCs cultured in the presence of CS, cells were *in vitro* cultured for 15 days with standard culture medium or chondrogenic differentiation medium. These cells were also cultured in the presence of different concentration of FBS or CS (2.5, 5, and 10%). The chondrogenic differentiation was then quantified using Alcian Blue staining, which indicates glycosaminoglycan depositions in the hAdMSCs. Our results showed that, when cultured with standard culture medium (in the absence of chondrogenic differentiation medium), no significant differences could be observed in those cells cocultured in presence of growing concentrations of CS when compared with equivalent concentrations of FBS (negative control) (**Figure 3A**). However, when cultured with a chondrogenic differentiation-specific medium, the glycosaminoglycan depositions were significantly higher in hAdMSCs cultured with the different concentration of CS compared with those with equivalent concentrations of FBS (positive control) (**Figure 3B**). In summary, these results demonstrate that under pro-chondrogenic differentiation conditions, the chondrocyte differentiation of hAdMSCs was significantly enhanced by CS.

### Concentration of Active and Latent TGF-β1 in Conditioned Serum

The concentration of both active and latent TGF-β1 in CS was determined by ELISA tests. The results obtained for active TGF-β1 showed a concentration of 65.99 pg/ml in 100% CS and 0.71 pg/ml in 10% CS (1:10 dilution). For latent TGF-β1, the concentrations were 125.06 ng/ml in 100% CS and 26.24 ng/ml in 1:10 diluted CS.

### Proliferative Behavior of *In Vitro*-Stimulated T Cells Cocultured in the Presence of Conditioned Serum-Sensitized Human Adipose-Derived Mesenchymal Stem Cells

**Figure 4:**
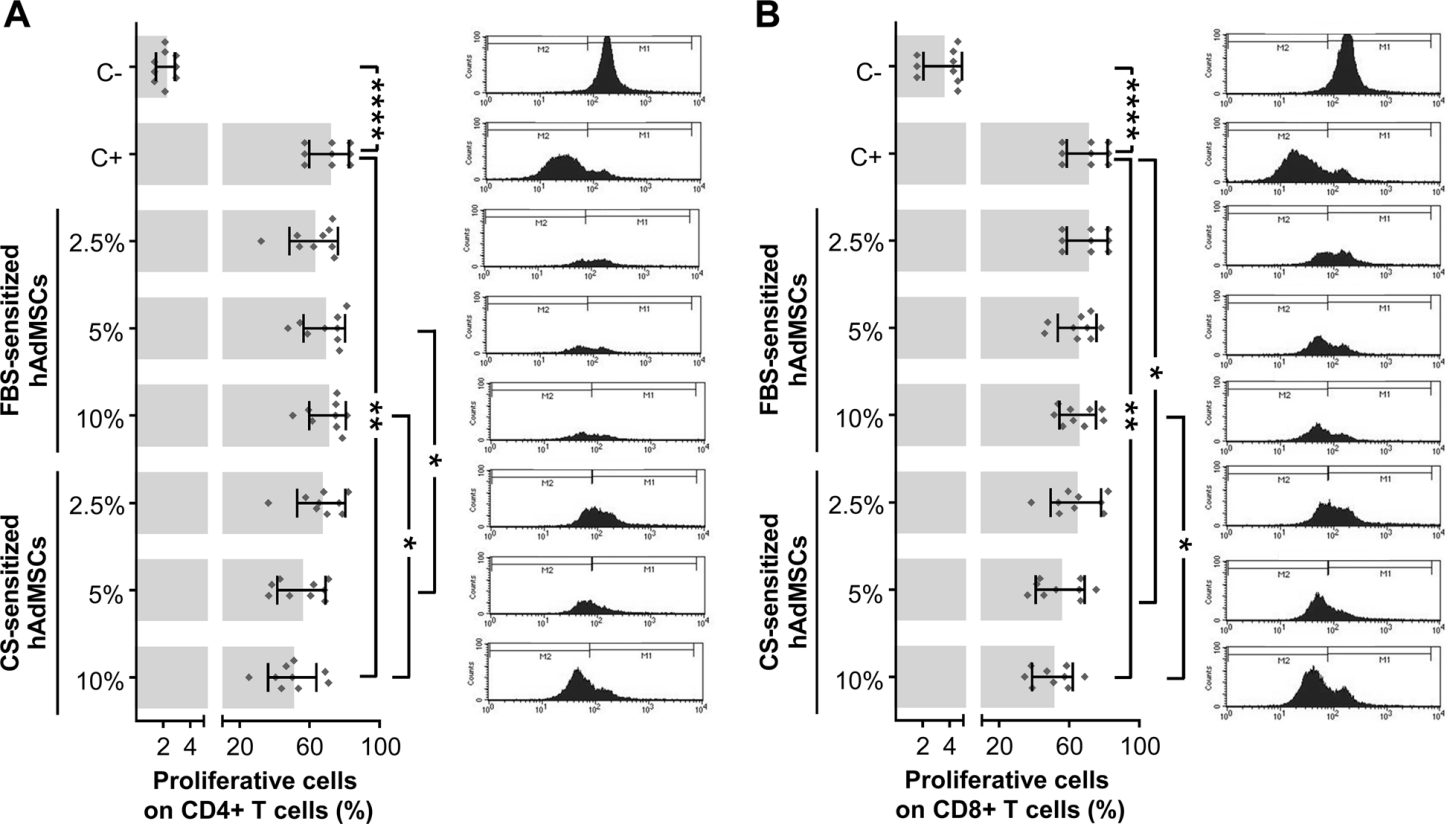
Proliferative ability of *in vitro*-stimulated T cells cocultured in the presence of FBS or CS-sensitized hAdMSCs. After 6 days of hAdMSCs sensitization with different concentrations (2.5, 5, and 10%) of FBS or CS in the culture medium (DMEM supplemented with 1% penicillin/streptomycin and 1% glutamine), these hAdMSCs (three different cell lines) were cocultured for 6 days with carboxyfluorescein succinimidyl ester (CFSE)-labeled peripheral blood lymphocytes (PBLs) (three different donors) during *in vitro* stimulation with T cell activation/expansion beads. As positive control (C+), stimulated PBLs without hAdMSCs were used, and as negative control (C−), non-stimulated PBLs were included. After coculture with hAdMSCs, PBLs were collected and stained with anti-CD4 and anti-CD8. The CFSE dilution assay allowed us to identify proliferative T cells (CFSE low) and non-proliferative T cells (CFSE high). Graphs representing individual values (*n* = 9), as well as mean ± SD of three independently performed experiments, together with representative histograms of each condition, are shown for CD4+ T cells **(A)** and CD8+ T cells **(B)**. Data were statistically analyzed using a one-way ANOVA test followed by a Fisher’s least significant difference test to compare equivalent concentrations of CS and FBS, C− with C+, and C+ with each treatment group. *p ≤ 0.05; **p ≤ 0.01; ****p ≤ 0.0001.

In order to study the hypothetical effect of CS on the immunomodulatory activity of hAdMSCs, we firstly aimed to determine the proliferative ability of *in vitro*-stimulated T cells cultured in the presence of CS-sensitized hAdMSCs. For that, CFSE-labeled PBLs were *in vitro* stimulated and cocultured for 6 days with 2.5, 5, and 10% FBS-sensitized or CS-sensitized hAdMSCs. The percentage of proliferative cells was calculated as the percentage of CFSE-low fluorescence intensity cells on gated CD4+ and CD8+ T cells. As shown in **Figure 4A**, the proliferation of CD4+ T cells was significantly reduced when PBLs were cocultured with 10% CS-sensitized hAdMSCs, compared with positive control. Also, significant differences were found when equivalent concentrations of FBS and CS were compared, observing that PBLs cocultured with 5 and 10% CS-sensitized hAdMSCs showed a significantly lower proliferation than their FBS counterparts. Similarly, as shown in **Figure 4B**, the proliferation of CD8+ T cells was significantly decreased when PBLs were cocultured in the presence of 5 and 10% CS-sensitized hAdMSCs, compared with the positive control, and also when cultured with 10% CS-sensitized hAdMSCs compared with 10% FBS-sensitized hAdMSCs.

### Differentiation of *In Vitro*-Stimulated T Cells Cocultured in the Presence of Conditioned Serum-Sensitized Human Adipose-Derived Mesenchymal Stem Cells

To continue with the study of the immunomodulatory activity of CS-sensitized hAdMSCs, the percentage of naïve cells (CD45RA+/CD62L+) was determined by flow cytometry on *in vitro*-stimulated T cells cultured in the presence of CS-sensitized hAdMSCs. For that, CFSE-labeled PBLs were *in vitro* stimulated and cocultured with 2.5, 5, and 10% FBS- or CS-sensitized hAdMSCs. After 6 days, PBLs were stained with fluorescence-labeled human mAbs against CD62L and CD45RA and analyzed by flow cytometry. The percentage of naïve cells was determined as the percentage of CD45RA+/CD62L+ cells on FSC/SSC-gated cells.

**Figure 5:**
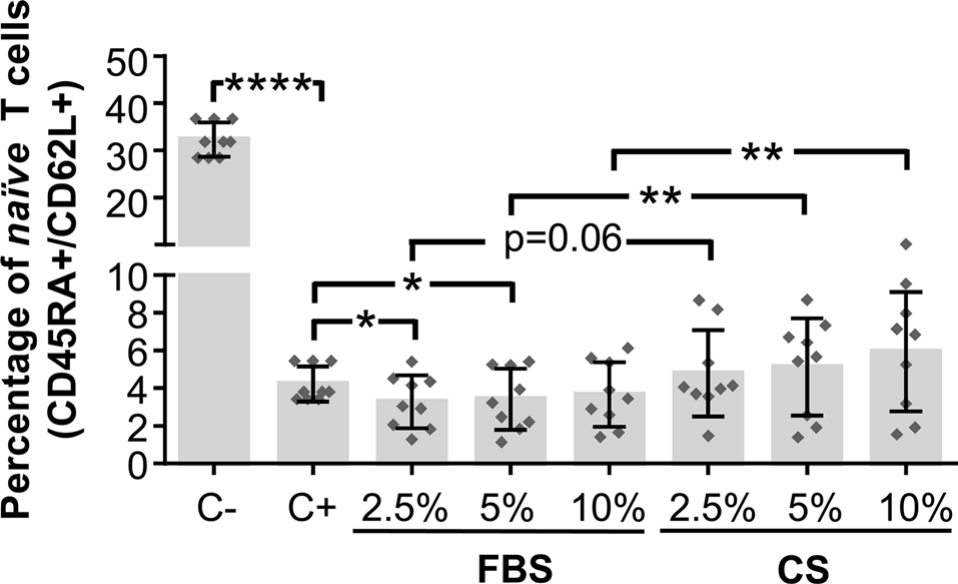
Activation and differentiation of *in vitro*-stimulated PBLs cultured in the presence of FBS or CS-sensitized hAdMSCs. After 6 days of hAdMSCs sensitization with different concentrations (2.5, 5, and 10%) of FBS or CS in the culture medium (DMEM supplemented with 1% penicillin/streptomycin and 1% glutamine), these hAdMSCs (three different cell lines) were cocultured for 6 days with PBLs (three different donors) during *in vitro* stimulation with T cell activation/expansion beads. As positive control (C+), stimulated PBLs without hAdMSCs were used, and as negative control (C−), non-stimulated PBLs were included. After coculture with hAdMSCs, CD45RA and CD62L co-expression was analyzed on PBLs by multicolor flow cytometry on FSC/SSC-gated cells. The graph shows individual values (*n* = 9) for the percentage of naïve T cells (CD45RA+/CD62L+), as well as mean ± SD of three independently performed experiments. Data were statistically analyzed using a one-way ANOVA test followed by a Fisher’s least significant difference test to compare equivalent concentrations of CS and FBS, C− with C+, and C+ with each treatment group. *p ≤ 0.05; **p ≤ 0.01; ****p ≤ 0.0001.

As expected, positive control (stimulated PBLs without hAdMSCs) showed a significant decrease of naïve T cells when compared with non-stimulated PBLs (negative control). Significant differences were found when comparing *in vitro*-stimulated PBLs cocultured with FBS-sensitized hAdMSCs and cocultured with CS-sensitized hAdMSCs. As shown in **Figure 5**, when PBLs were cocultured with 2.5 and 5% FBS-sensitized hAdMSCs, the percentage of naïve cells significantly decreased in comparison with positive control. Additionally, no statistically significant differences were found when comparing the different treatment groups with the positive control. Interestingly, there were significant increases in the percentage of naïve cells in those PBLs cocultured with 5 and 10% CS-sensitized hAdMSCs when compared with their FBS counterparts. These results suggest that CS sensitization in hAdMSCs induced a higher immunomodulatory effect of these stem cells against T cell activation.

## Discussion

Osteoarthritis is the most common form of arthritis, representing a major cause of disability, especially in elderly patients and causing an ever growing burden for health care systems (Mobasheri and Henrotin, 2015). Osteoarthritis is characterized by progressive cartilage degeneration, and emerging therapies are focused in the activation of cartilage regenerative potential to slow its degeneration and preserving joint function (Li et al., 2017). Osteoarthritis has been hypothesized to be a “chronic wound”-type disease (Scanzello et al., 2008; Wu and Henry, 2012) characterized among others by a chronically upregulated innate immune system. Interestingly, similar mechanisms are observed in chronic tendon injuries (Klatte-Schulz et al., 2018). These processes—far from perfectly understood—may have a major impact not only on osteoarthritis degenerative pathology but also on pain perception.

CS is a practical and relatively inexpensive method to generate IL-1Ra (Evans et al., 2016). There is a consensus in the literature that suggests that CS can reduce degenerative mechanisms and enhance tissue regeneration (Frizziero et al., 2013). Moreover, it is widely accepted that the local treatment is a safe and effective therapy (Fotouhi et al., 2018).

The aim of this work was to evaluate—under *in vitro* culture conditions—the effect of CS on the chondrogenic and immunomodulatory potential of hAdMSCs. Both CS (which contains anti-inflammatory cytokines and growth factors) and hAdMSCs (with a potent anti-inflammatory effect) are currently considered as some of the most promising emerging treatments for cartilage repair (de Girolamo et al., 2016). However, at present, there are no studies that explore the combination of these two therapies.

Our study was firstly conducted to evaluate the phenotype, viability, and proliferative behavior of hAdMSCs cultured in the presence of CS. Our results showed that the phenotypic profile and viability of hAdMSCs cocultured with CS were similar or equivalent to hAdMSCs cultured under standard culture conditions (supplemented with FBS). Uniquely, CD90 was increased in the CS groups, which could be directly related to the cell confluence (Dudakovic et al., 2014). These results suggest that, in future clinical trials aiming to combine CS and hAdMSCs, CS could be considered as an optimal delivery vehicle for stem cell administration.

Once demonstrated that CS does not exhibit any adverse effect on hAdMSCs, we aimed to determine the chondrogenic potential of hAdMSCs in the presence of CS. Interestingly, the chondrogenic differentiation of hAdMSCs was significantly enhanced by CS in the presence of a specific differentiation medium. These results may have a clinical relevance in stem cell-based therapies using CS as a vehicle and suggest that CS may improve the chondrogenic potential of implanted stem cells. Further studies should be conducted to determine if the therapeutic effect of CS (in terms of cartilage reparation) in osteoarthritis patients could be mediated, at least in part, by the chondrogenic activation of resident stem cells after intra-articular injections of CS. Interestingly, a muscle contusion mouse model showed increased numbers of satellite cells in the healing tissue after local CS injection vs saline, and clinically, this finding correlated to a significantly faster recovery of muscle injuries in athletes (Wright-Carpenter et al., 2004).

Although the identification of soluble factors involved in the chondrogenic activation of hAdMSCs is not the main issue of this paper, we hypothesize that the presence of TGF-β1 in CS could be a major mediator for the enhancement of chondrogenic differentiation. The role of TGF-β1 in the chondrogenic differentiation has been widely described in different cell lines such as MSCs (Cals et al., 2012), chondrocytes (Murphy et al., 2015), and human synovium-derived stem cells (Kim et al., 2014). Previous studies have also demonstrated that TGF-β1 is significantly increased in CS (Rutgers et al., 2010). Similarly to Rutgers et al., our quantitative analyses of TGF-β1 demonstrated that both active and latent TGF-β were abundant in CS (>50 pg/ml of active TGF-β1 and >100 ng/ml of latent TGF-β1). The hypothesis of TGF-β involvement in the chondrogenic activation of hAdMSCs could also be supported by previous studies that demonstrated that total TGF-β in CS from 80 patients was very high (97.93 ± 113.41 ng/ml) (Wehling et al., 2007). In contrast, although FBS contains total TGF-β (10–20 ng/ml), its concentration is about 10 times lower than in CS (Oida and Weiner, 2010).

Finally, it has been recently reported that IGF-1 (also present in CS) plays a role in the chondrogenic differentiation (Zhou et al., 2016), so we should not discard that the chondrogenic response of stem cells could also be mediated by IGF-1.

On the other hand, equivalent results on activated PRP have also been described. In a recent work published by Van Pham et al., the authors showed that activated PRP promoted the proliferation of hAdMSCs as well as their differentiation toward chondrocytes (Van Pham et al., 2013). Similarly, adipose- and bone marrow-derived stem cells seeded in PRP-derived scaffolds showed a higher proliferative rate and chondrocyte differentiation when compared with control cells (Xie et al., 2012). These reports suggest that both CS and PRP are providing a suitable environment for stem cell proliferation and differentiation toward chondrogenic lineage.

Regarding the differences between PRP and CS, in terms of soluble factors, the main difference has been previously described for IL-1Ra, which is high in CS but almost absent in PRP (Wehling et al., 2007; Mussano et al., 2016). This may explain the differences between CS and PRP in terms of duration of the achieved positive effects and the positive impact on the arthrologic status (Shirokova et al., 2017). Another issue with PRP upon intra-articular injection is the sudden presence of a high number of cells that originally do not belong to the joint. Both platelets and leukocytes may—in the presence of appropriate stimulus—act as amplifiers of innate immune reactions leading to transient intense pain in 1 out of 10 patients (Standford University Medical Center—Department of Radiology).

Considering that the therapeutic effect of adult stem cells has been attributed to their immunomodulatory effect in the inflammatory process, our experiments were conducted to evaluate the immunomodulatory capacity of hAdMSCs sensitized with CS. This immunomodulatory capacity was evaluated against *in vitro*-stimulated T cells using anti-CD2, anti-CD3, and anti-CD28 that partially mimic the stimulation by antigen-presenting cells (Trickett and Kwan, 2003). Based on previous methodologies from our group (Blazquez et al., 2014; Álvarez et al., 2018), the immunomodulation was assessed by measuring the proliferative behavior of T cells and the percentage of naïve T cells according to the co-expression of CD45RA and CD62L (Foster et al., 2004; Prabhu et al., 2016).

Our results showed that CS-sensitized hAdMSCs significantly decreased the proliferation and differentiation potential of *in vitro*-stimulated T cells. Although previous studies have reported that the immunomodulatory ability of MSCs can be triggered by different cytokines such as IFN-γ, TNF-α, IL-1β, or IL-17A (Prasanna et al., 2010; Gray et al., 2015; Mohammadpour et al., 2015; Sivanathan et al., 2015), here, we hypothesize that TGF-β1 may trigger this immunomodulatory effect on hAdMSCs. Supporting this hypothesis, de Witte et al. have previously shown on umbilical cord MSCs that TGF-β1-sensitized MSCs were able to significantly reduce T cell proliferation (de Witte et al., 2017). Finally, apart from TGF-β1, which has been also associated to MSCs migration and proliferation (Sun et al., 2013; Zhang et al., 2015; Tyurin-Kuzmin et al., 2016), the presence of different growth factors in CS such as VEGF or HGF has been demonstrated to be a key factor for the therapeutic effect of MSCs (Deuse et al., 2009; Tögel et al., 2009; Zisa et al., 2009).

To our knowledge, this is the first *in vitro* study that analyzes the combination of CS and hAdMSCs. Here, we demonstrate that CS enhances the chondrogenic potential and immunomodulatory activity of hAdMSCs, suggesting that CS pre-sensitization or *in vivo* coadministration may have a beneficial effect on the therapeutic potential of hAdMSCs. Moreover, these results suggest that intra-articular administration of CS might influence the biological behavior of resident stem cells, increasing their chondrogenic differentiation and immunomodulatory activity.

## Author Contributions

RB, FS, and JC conceived and designed the experiments. VA, RB, and JC performed the experiments and analyzed the data. RB, FS, JR, and JC wrote the manuscript. EL and FM revised the statistical analysis, English grammar, manuscript discussion, and revision. All authors read and approved the final manuscript.

## Funding

This work was supported in part by Zaraclinics S.L.; ISCIII grants (CP17/00021, MS17/00021, PI18/0911 to JGC), co-funded by ERDF/ESF, “Investing in your future”; one grant from Consejería de Economía e Infraestructuras, Junta de Extremadura co-funded by ERDF (IB16168 to JGC); one grant from GobEx (Ayuda a grupos catalogados de la Junta de Extremadura, GR18199) and by CIBERCV (CB16/11/00494 to FMSM). The funders had no role in study design, data collection, analysis and interpretation, and decision to publish or preparation of the manuscript.

## Conflict of Interest Statement

The author JR is an employee of Orthogen AG.

The remaining authors declare that this research was conducted in the absence of any commercial or financial relationships that could be construed as a potential conflict of interest.
