# Supplementary figures and images for: Conditioned Serum Enhances the Chondrogenic and Immunomodulatory Behavior of Mesenchymal Stem Cells
Source: Front Pharmacol. 2019 Jun 28;10:699. doi: 10.3389/fphar.2019.00699 (PMC6609570; doi:10.3389/fphar.2019.00699)

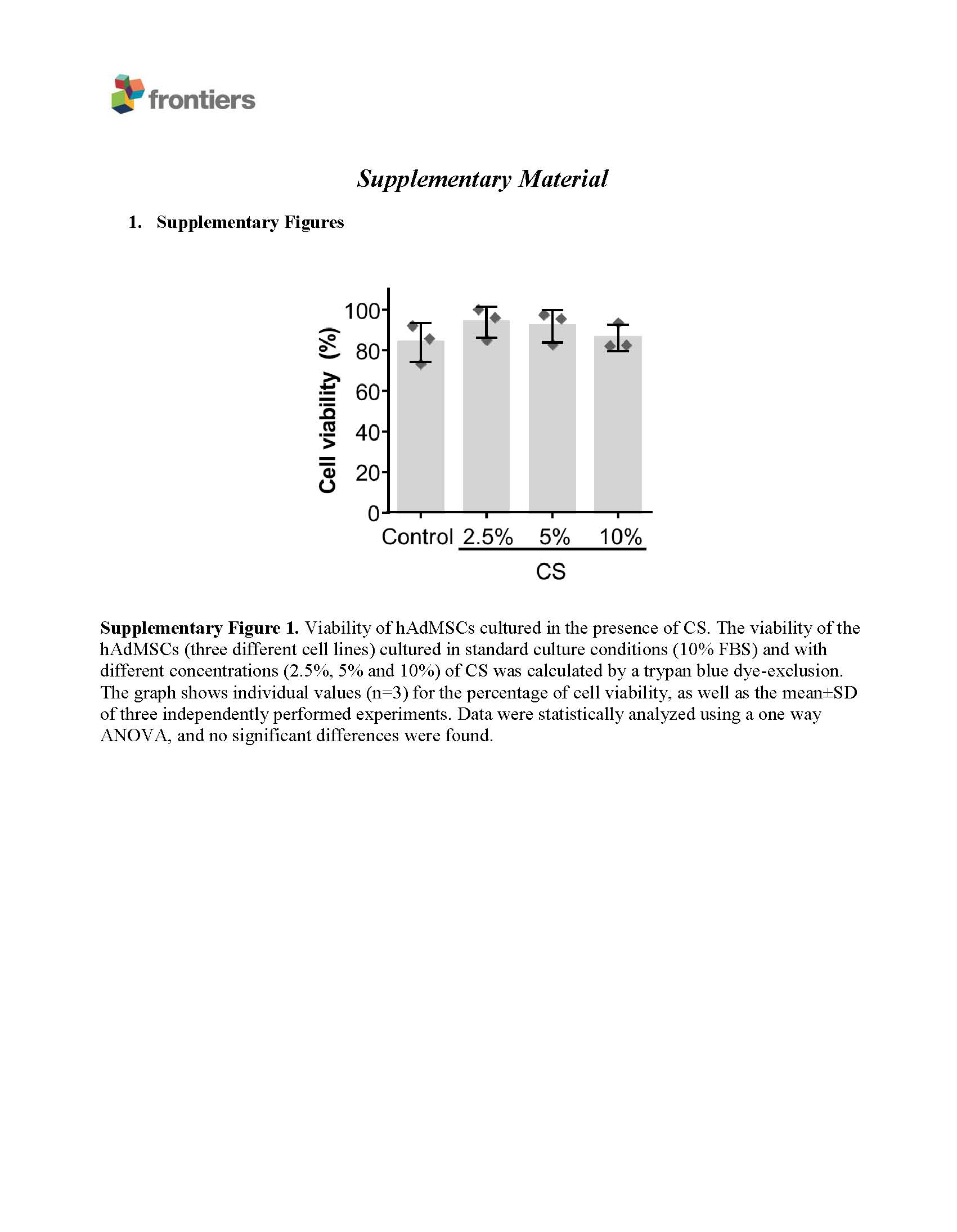

Supplement: Supplementary file 1 [file Image_1.tif]
